# The top fifty most influential articles on hip fractures

**DOI:** 10.1007/s00264-022-05511-0

**Published:** 2022-07-23

**Authors:** Gilbert Manuel Schwarz, Stefan Hajdu, Reinhard Windhager, Madeleine Willegger

**Affiliations:** Department of Orthopedics and Trauma Surgery, Medical University of Vienna, Waehringer Guertel 18-20, 1090 Vienna, Austria

**Keywords:** Hip fracture, Perioperative management, Citation analysis, Bibliographic analysis

## Abstract

**Purpose:**

Hip fractures are one of the most common disabling fractures in elderly people and peri-operative management has advanced considerably over the past decades. The purpose of this study was to evaluate the change of scientific focus by creating a top 50 list of the most influential papers on this topic.

**Methods:**

The *Clarivate Web of Science Search* was used to identify the most cited articles. The used search phrase was [(hip OR pertrochanteric OR (femoral neck)) AND fracture AND (surgery OR treatment)]. The number of citations, citation density, study type, study design, published year, fracture type, country, evidence level and published journal were recorded.

**Results:**

The top 50 articles were published between 1973 and 2014 and cited between 88 and 496 times. The mean citation density increased noticeably after the year 2000, representing the knowledge gain of the last 20 years. The topics *surgical treatment* (*n* = 19), *risk factor assessment* (*n* = 19), *perioperative hemodynamic management* (*n* = 7), *additional treatment* (*n* = 4) and *general reviews* (*n* = 1) were covered. Twenty-five articles were published from institutions in Europe, 24 from institutions in North America and one from an institution in Asia.

**Conclusion:**

While studies about surgical treatment options and risk factor assessment have been historically important, there was a rise of articles about additional treatment options for osteoporosis and the optimal postoperative care after the year 2005. The presented lists and map of citation classics give an overview of the most influential studies on hip fractures.

## Introduction

Hip fractures are one of the most common fractures in elderly patients [1]. The one year mortality ranges between 14 and 36% [2, 3]. In 2000, more than 1.6 million hip fractures occurred globally and accounted for 20% of all fractures in patients over 50 years [4]. It is estimated that the absolute number of annual fractures will be 4.5 million by the year 2050 [5, 6]. Hip fractures are among the classic fragility fractures of geriatric patients and more than 90% are caused by low energy trauma (i.e. fall from standing height). Established risk factors are osteoporosis, high age, female sex, smoking and a low BMI [4, 7–9]. They can be classified into femoral neck fractures, per- or intertrochanteric fractures and subtrochanteric fractures [10]. While per- or intertrochanteric fractures are treated with osteosynthesis devices, femoral neck fractures can be either treated with hemi- and total hip arthroplasty or osteosynthesis [11].

The enormous prevalence of hip fractures accentuates the socio-economic significance and explains the sheer infinite number of published articles [12]. In an era of evidence-based medicine, research studies are not only important for a better understanding but also in clinical decision-making. With the increase in studies published recently, it is becoming difficult to overlook the most current research questions. One way to determine the impact of a published article is to use the citation analysis [13–18]. Although the quality of an article does not depend solely on its citation rate, it represents its importance in the field and is widely recognized in the scientific community.

The aim of this study was to identify the top 50 most influential articles on hip fractures. To characterize the change of scientific focus and research questions in recent years, the top ten articles over the last five years (2015–2020) were separately evaluated. It was hypothesized that the literature on hip fracture treatment would change over the decades, as the evidence base and quality of studies were expected to improve over time.

## Material and methods

### Search strategy

The Clarivate Web of Science search was used to identify the most cited articles regarding hip fractures. The used search phrase was [(hip OR pertrochanteric OR (femoral neck)) AND fracture AND (surgery OR treatment)]. It was performed on 18th March 2020 and no institutional review board or ethical approval was required. The options “All databases”, “Basic Search”, “All years” and search based on the “topic” were applied. The first 100 articles were thoroughly studied and excluded (1) if hip fractures were not the main topic and (2) if there was no full text available.

**Fig. 1:**
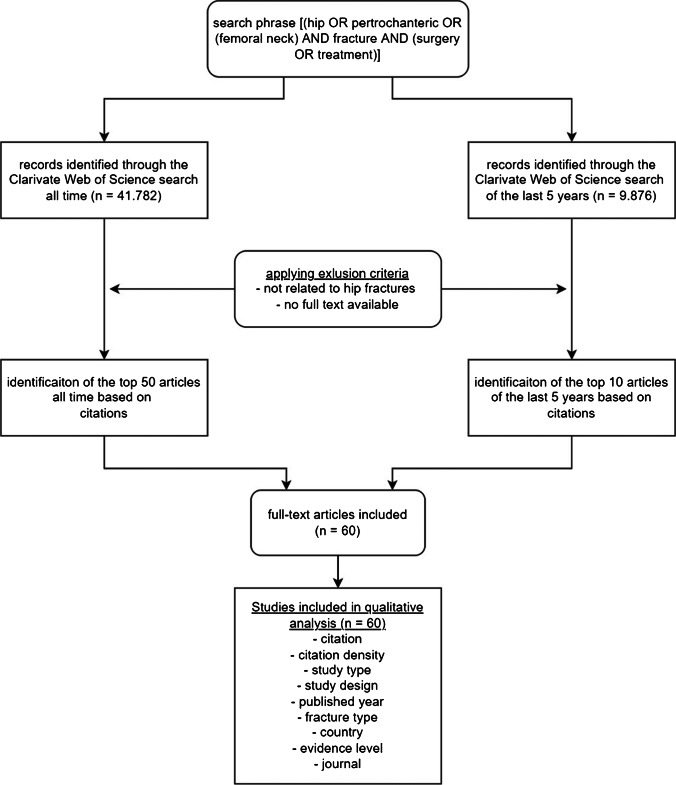
Flowchart of the search algorithm. The used search phrase was [(hip OR pertrochanteric OR (femoral neck)) AND fracture AND (surgery OR treatment)]. The top articles of all time (left) and the top articles from 2015 to 2020 (right) were evaluated

A second search was performed for articles published over the last five years (2015–2020). With the exception of the time frame, the same parameters as described above were used. Finally, 60 articles were included for the final analysis. Data extraction was performed according to an adapted PRISMA flowchart (Fig. 1). Because no patients were involved, ethics committee approval was waived for this cross-sectional study.

### Qualitative analysis

All articles were organized in descending order according to overall number of citations. Two different tables were created: the first for the top 50 articles of all time (Table 1) and the second for the top ten articles between 2015 and 2020 (Table 2). The following data were extracted from all articles: overall number of citations, citation density, level of evidence, title, first authors name, senior authors name, publication year, published journal, country, institution (according to corresponding author) and language. The level of evidence was either acquired from the article itself or assigned according to the Practical Guide from Wright [19]. In case of questionable results, evidence levels, study types and study designs were clarified in consensus meetings with the senior author.

**Table 1:** Top 50 articles published worldwide

No	Article name	No. of citations (citation density)	Topic	Study design	Evidence level
1	Eriksson BI, Bauer KA, Lassen MR, Turpie AGG, Steering Comm Pentasaccharide H. Fondaparinux compared with enoxaparin for the prevention of venous thromboembolism after hip-fracture surgery. N Engl J Med. 2001;345:1298–304	496 (24.8)	Hemodynamic management	Randomized controlled trial	II
2	Moran CG, Wenn RT, Sikand M, Taylor AM. Early mortality after hip fracture: Is delay before surgery important? Journal of Bone and Joint Surgery-American Volume. 2005;87A:483–9	413 (25.8)	Risk factor assessment	Prospective cohort study	II
3	Kenzora JE, McCarthy RE, Lowell JD, Sledge CB. Hip fracture mortality. Relation to age, treatment, preoperative illness, time of surgery, and complicationsClin Orthop Relat Res. 1984:45–56	379 (10.2)	Risk factor assessment	Retrospective case series	IV
4	Simunovic N, Devereaux PJ, Sprague S, Guyatt GH, Schemitsch E, DeBeer J, et al. Effect of early surgery after hip fracture on mortality and complications: systematic review and meta-analysis. Can Med Assoc J. 2010;182:1609–16	352 (32)	Risk factor assessment	Systematic review and meta-analysis	IV
5	Orosz GM, Magaziner J, Hannan EL, Morrison RS, Koval K, Gilbert M, et al. Association of timing of surgery for hip fracture and patient outcomes. Jama-Journal of the American Medical Association. 2004;291:1738–43	328 (19.3)	Risk factor assessment	Prospective cohort study	II
6	Eriksson BI, Lassen MR, Inv PP. Duration of prophylaxis against venous thromboembolism with fondaparinux after hip fracture surgery—A multicenter, randomized, placebo-controlled, double-blind study. Arch Intern Med. 2003;163:1337–42	290 (16.1)	Hemodynamic management	Randomized controlled trial	I
7	Keating JF, Grant A, Masson N, Scott NW, Forbes JF, Scottish Orthopaedic Trials N. Randomized comparison of reduction and fixation, bipolar hemiarthroplasty, and total hip arthroplasty—Treatment of displaced intracapsular hip fractures in healthy older patients. Journal of Bone and Joint Surgery-American Volume. 2006;88A:249–60	277 (18.5)	Surgical treatment	Randomized controlled trial	II
8	Hu FK, Jiang CY, Shen J, Tang PF, Wang Y. Preoperative predictors for mortality following hip fracture surgery: A systematic review and meta-analysis. Injury-International Journal of the Care of the Injured. 2012;43:676–85	263 (26.3)	Risk factor assessment	Systematic review and meta-analysis	IV
9	Anglen JO, Weinstein JN, American Board of Orthopaedic Surgery Research C. Nail or plate fixation of intertrochanteric hip fractures: changing pattern of practice. A review of the American Board of Orthopaedic Surgery Database. J Bone Joint Surg Am. 2008;90:700–7	253 (19.5)	Surgical treatment	Review	IV
10	Moja L, Piatti A, Pecoraro V, Ricci C, Virgili G, Salanti G, et al. Timing Matters in Hip Fracture Surgery: Patients Operated within 48 Hours Have Better Outcomes. A Meta-Analysis and Meta-Regression of over 190,000 Patients. PLoS One. 2012;7	217 (24.1)	Risk factor assessment	Systematic review and meta-analysis	III
11	Khan SK, Kalra S, Khanna A, Thiruvengada MM, Parker MJ. Timing of surgery for hip fractures: A systematic review of 52 published studies involving 291,413 patients. Injury-International Journal of the Care of the Injured. 2009;40:692–7	205 (17.1)	Risk factor assessment	Systematic review and meta-analysis	III
12	Haidukewych GJ, Rothwell WS, Jacofsky DJ, Torchia ME, Berry DJ. Operative treatment of femoral neck fractures in patients between the ages of fifteen and fifty years. Journal of Bone and Joint Surgery-American Volume. 2004;86A:1711–6	193 (11.4)	Surgical treatment	Retrospective cohort study	IV
13	Grimes JP, Gregory PM, Noveck H, Butler MS, Carson JL. The effects of time-to-surgery on mortality and morbidity in patients following hip fracture. Am J Med. 2002;112:702–9	192 (10.1)	Risk factor assessment	Retrospective cohort study	IV
14	Foss NB, Kehlet H. Hidden blood loss after surgery for hip fracture. Journal of Bone and Joint Surgery-British Volume. 2006;88B:1053–9	190 (12.7)	Hemodynamic management	Case series	IV
15	Powers PJ, Gent M, Jay RM, Julian DH, Turpie AGG, Levine M, et al. A randomized trial of less intense postoperative warfarin or aspirin therapy in the prevention of venous thromboembolism after surgery for fractured hip. Arch Intern Med. 1989;149:771–4	188 (5.9)	Hemodynamic management	Randomized controlled trial	II
16	Madsen JE, Naess L, Aune AK, Alho A, Ekeland A, Stromsoe K. Dynamic hip screw with trochanteric stabilizing plate in the treatment of unstable proximal femoral fractures: A comparative study with the Gamma nail and compression hip screw. J Orthop Trauma. 1998;12:241–8	179 (7.8)	Surgical treatment	Randomized controlled trial	II
17	Johansson T, Jacobsson SA, Ivarsson I, Knutsson A, Wahlstrom O. Internal fixation versus total hip arthroplasty in the treatment of displaced femoral neck fractures—A prospective randomized study of 100 hips. Acta Orthop Scand. 2000;71:597–602	162 (7.7)	Surgical treatment	Randomized controlled trial	II
18	Baumgaertner MR, Curtin SL, Lindskog DM. Intramedullary versus extramedullary fixation for the treatment of intertrochanteric hip fractures. Clin Orthop Relat Res. 1998:87–94	160 (7.0)	Surgical treatment	Randomized controlled trial	II
19	Lefaivre KA, Macadam SA, Davidson DJ, Gandhi R, Chan H, Broekhuyse HM. Length of stay, mortality, morbidity and delay to surgery in hip fractures. Journal of Bone and Joint Surgery-British Volume. 2009;91B:922–7	157 (13.1)	Risk factor assessment	Retrospective cohort study	IV
20	Luyao GL, Baron JA, Barrett JA, Fisher ES. Treatment and survival among elderly americans with hip-fractures—a population-based study. Am J Public Health. 1994;84:1287–91	156 (6)	Risk factor assessment	Cross-sectional study	IV
21	Weller I, Wai EK, Jaglal S, Kreder HJ. The effect of hospital type and surgical delay on mortality after surgery for hip fracture. Journal of Bone and Joint Surgery-British Volume. 2005;87B:361–6	146 (10.4)	Risk factor assessment	Retrospective cohort study	IV
22	Haidukewych GJ, Berry DJ. Hip arthroplasty for salvage of failed treatment of intertrochanteric hip fractures. Journal of Bone and Joint Surgery-American Volume. 2003;85A:899–904	144 (8)	Surgical treatment	Retrospective case series	IV
23	Barton TM, Gleeson R, Topliss C, Greenwood R, Harries WJ, Chesser TJS. A Comparison of the Long Gamma Nail with the Sliding Hip Screw for the Treatment of AO/OTA 31-A2 Fractures of the Proximal Part of the Femur A Prospective Randomized Trial. Journal of Bone and Joint Surgery-American Volume. 2010;92A:792–8	140 (12.7)	Surgical treatment	Non-randomized controlled trial	I
24	Gardner MJ, Brophy RH, Demetrakopoulos D, Koob J, Hong R, Rana A, et al. Interventions to improve osteoporosis treatment following hip fracture—A prospective, randomized trial. Journal of Bone and Joint Surgery-American Volume. 2005;87A:3–7	140 (8.8)	Additional treatment	Randomized controlled trial	I
25	Dorr LD, Glousman R, Hoy ALS, Vanis R, Chandler R. Treatment of Femoral Neck Fractures With Total Hip Replacement Versus Cemented And Noncemented Hemiarthroplasty. The Journal of arthroplasty. 1986;1:21–8	140 (4)	Surgical treatment	Non-randomized controlled trial	II
26	Maxwell MJ, Moran CG, Moppett IK. Development and validation of a preoperative scoring system to predict 30 day mortality in patients undergoing hip fracture surgery. Br J Anaesth. 2008;101:511–7	138 (10.6)	Risk factor assessment	Prospective cohort study	I
27	Smith T, Pelpola K, Ball M, Ong A, Myint PK. Pre-operative indicators for mortality following hip fracture surgery: a systematic review and meta-analysis. Age Ageing. 2014;43:464–71	135 (19.3)	Risk factor assessment	Systematic review and meta-analysis	IV
28	Strauss E, Frank J, Lee J, Kummer FJ, Tejwani N. Helical blade versus sliding hip screw for treatment of unstable intertrochanteric hip fractures: A biomechanical evaluation. Injury-International Journal of the Care of the Injured. 2006;37:984–9	134 (8.9)	Surgical treatment	Basic science	II
29	Swiontkowski MF, Hansen ST, Kellam J. Ipsilateral fractures of the femoral neck and shaft. A treatment protocol. Journal of Bone and Joint Surgery-American Volume. 1984;66A:260–8	132 (3.6)	Surgical treatment	Case series	IV
30	Hamlet WP, Lieberman JR, Freedman EL, Dorey FJ, Fletcher A, Johnson EE. Influence of health status and the timing of surgery on mortality in hip fracture patients. Am J Orthop (Belle Mead NJ). 1997;26:621–7	130 (5.4)	Risk factor assessment	Retrospective case series	IV
31	Darcy J, Devas M. Treatment of fractures of the femoral neck by replacement with the Thompson prosthesis. Journal of Bone and Joint Surgery-British Volume. 1976;58:279–86	129 (2.9)	Surgical treatment	Retrospective case series	IV
32	Kamel HK, Iqbal MA, Mogallapu R, Maas D, Hoffmann RG. Time to ambulation after hip fracture surgery: Relation to hospitalization outcomes. Journals of Gerontology Series a-Biological Sciences and Medical Sciences. 2003;58:1042–5	124 (6.9)	Risk factor assessment	Retrospective cohort study	III
33	Novack V, Jotkowitz A, Etzion O, Porath A. Does delay in surgery after hip fracture lead to worse outcomes? A multicenter survey. Int J Qual Health Care. 2007;19:170–6	122 (8.7)	Risk factor assessment	Retrospective case series	IV
34	Goodman SB, Bauer TW, Carter D, Casteleyn PP, Goldstein SA, Kyle RF, et al. Norian SRS cement augmentation in hip fracture treatment—Laboratory and initial clinical results. Clin Orthop Relat Res. 1998:42–50	120 (5.2)	Surgical treatment	Case series	IV
35	Edwards C, Counsell A, Boulton C, Moran CG. Early infection after hip fracture surgery—Risk factors, costs and outcome. Journal of Bone and Joint Surgery-British Volume. 2008;90B:770–7	116 (8.9)	Risk factor assessment	Retrospective cohort study	III
36	Zufferey PJ, Miquet M, Quenet S, Martin P, Adam P, Albaladejo P, et al. Tranexamic acid in hip fracture surgery: a randomized controlled trial. Br J Anaesth. 2010;104:23–30	113 (10.3)	Hemodynamic management	Randomized controlled trial	II
37	Foss NB, Kristensen MT, Jensen PS, Palm H, Krasheninnikoff M, Kehlet H. The effects of liberal versus restrictive transfusion thresholds on ambulation after hip fracture surgery. Transfusion. 2009;49:227–34	112 (9.3)	Hemodynamic management	Randomized controlled trial	II
38	Macaulay W, Nellans KW, Garvin KL, Iorio R, Healy WL, Rosenwasser MP, et al. Prospective randomized clinical trial comparing hemiarthroplasty to total hip arthroplasty in the treatment of displaced femoral neck fractures—Winner of the Dorr Award. J Arthroplasty. 2008;23:2–8	110 (8.5)	Surgical treatment	Randomized controlled trial	II
39	Foss NB, Kristensen MT, Kehlet H. Anaemia impedes functional mobility after hip fracture surgery. Age Ageing. 2008;37:173–8	109 (8.4)	Hemodynamic management	Non-randomized controlled trial	II
40	Lee BPH, Berry DJ, Harmsen WS, Sim FH. Total hip arthroplasty for the treatment of an acute fracture of the femoral neck—Long-term results. Journal of Bone and Joint Surgery-American Volume. 1998;80A:70–5	105 (4.6)	Surgical treatment	Retrospective case series	IV
41	Meyers MH, Harvey JP, Moore TM. Treatment of displaced subcapital and transcervical fractures of the femoral neck by muscle-pedicle-bone graft and internal fixation. A preliminary report on one hundred and fifty cases . Journal of Bone and Joint Surgery-American Volume. 1973;A 55:257–74	104 (2.2)	Surgical treatment	Case series	IV
42	Gjertsen JE, Vinje T, Engesaeter LB, Lie SA, Havelin LI, Furnes O, et al. Internal Screw Fixation Compared with Bipolar Hemiarthroplasty for Treatment of Displaced Femoral Neck Fractures in Elderly Patients. Journal of Bone and Joint Surgery-American Volume. 2010;92A:619–28	103 (9.4)	Surgical treatment	Retrospective cohort study	III
43	Jennings LA, Auerbach AD, Maselli J, Pekow PS, Lindenauer PK, Lee SJ. Missed Opportunities for Osteoporosis Treatment in Patients Hospitalized for Hip Fracture. J Am Geriatr Soc. 2010;58:650–7	101 (9.2)	Additional treatment	Retrospective cohort study	IV
44	Rabenda V, Vanoverloop J, Fabri V, Mertens R, Sumkay F, Vannccke C, et al. Low Incidence of Anti-Osteoporosis Treatment After Hip Fracture. Journal of Bone and Joint Surgery-American Volume. 2008;90A:2142–8	100 (7.7)	Additional treatment	Retrospective cohort study	IV
45	Banan H, Al-Sabti A, Jimulia T, Hart AJ. The treatment of unstable, extracapsular hip fractures with the AO/ASIF proximal femoral nail (PFN)—our first 60 cases. Injury-International Journal of the Care of the Injured. 2002;33:401–5	96 (5.1)	Surgical treatment	Retrospective cohort study	IV
46	McGuire KJ, Bernstein J, Polsky D, Silber JH. The 2004 Marshall Urist Award—Delays until surgery after hip fracture increases mortality. Clin Orthop Relat Res. 2004:294–301	91 (5.4)	Risk factor assessment	Retrospective cohort study	IV
47	Uzoigwe CE, Burnand HGF, Cheesman CL, Aghedo DO, Faizi M, Middleton RG. Early and ultra-early surgery in hip fracture patients improves survival. Injury-International Journal of the Care of the Injured. 2013;44:726–9	90 (11.3)	Risk factor assessment	Retrospective case series	IV
48	Holmberg S, Kalen R, Thorngren KG. Treatment and outcome of femoral neck fractures. An analysis of 2418 patients admitted from their own homes. Clin Orthop Relat Res. 1987:42–52	90 (2.6)	Surgical treatment	Retrospective cohort study	III
49	Hommel A, Ulander K, Bjorkelund KB, Norrman PO, Wingstrand H, Thorngren KG. Influence of optimised treatment of people with hip fracture on time to operation, length of hospital stay, reoperations and mortality within 1 year. Injury-International Journal of the Care of the Injured. 2008;39:1164–74	89 (3.9)	Additional treatment	Prospective cohort study	II
50	Lyons AR. Clinical outcomes and treatment of hip fractures. Am J Med. 1997;103:51S-63S; discussion S-4S	88 (3.8)	General review	Review	V

**Table 2:** Top 10 articles between 2015 and 2020 worldwide

No	Article name	No. of citations (citation density)	Topic	Study design	Evidence level
1	Pincus D, Ravi B, Wasserstein D, Huang A, Paterson JM, Nathens AB, et al. Association Between Wait Time and 30-Day Mortality in Adults Undergoing Hip Fracture Surgery. JAMA. 2017;318:1994–2003	74 (18.5)	Risk factor assessment	Retrospective cohort study	III
2	Rogmark C, Leonardsson O. Hip arthroplasty for the treatment of displaced fractures of the femoral neck in elderly patients. Bone Joint J. 2016;98-B:291–7	52 (10.4)	Surgical treatment	Review	IV
3	Nauth A, Creek AT, Zellar A, Lawendy A-R, Dowrick A, Gupta A, et al. Fracture fixation in the operative management of hip fractures (FAITH): an international, multicentre, randomised controlled trial. The Lancet. 2017;389:1519–27	39 (9.8)	Surgical treatment	Randomized controlled trial	II
4	Sheikh HQ, Hossain FS, Aqil A, Akinbamijo B, Mushtaq V, Kapoor H. A Comprehensive Analysis of the Causes and Predictors of 30-Day Mortality Following Hip Fracture Surgery. Clin Orthop Surg. 2017;9:10–8	30 (7.5)	Risk factor assessment	Retrospective case series	IV
5	Socci AR, Casemyr NE, Leslie MP, Baumgaertner MR. Implant options for the treatment of intertrochanteric fractures of the hip: rationale, evidence, and recommendations. Bone Joint J. 2017;99-B:128–33	30 (7.5)	Surgical treatment	Review	V
6	Kilci O, Un C, Sacan O, Gamli M, Baskan S, Baydar M, et al. Postoperative Mortality after Hip Fracture Surgery: A 3 Years Follow Up. PLoS One. 2016;11:e0162097	28 (5.6)	Risk factor assessment	Retrospective case series	IV
7	Forni S, Pieralli F, Sergi A, Lorini C, Bonaccorsi G, Vannucci A. Mortality after hip fracture in the elderly: The role of a multidisciplinary approach and time to surgery in a retrospective observational study on 23,973 patients. Arch Gerontol Geriatr. 2016;66:13–7	28 (5.6)	Additional treatment	Retrospective cohort study	IV
8	Folbert EC, Hegeman JH, Vermeer M, Regtuijt EM, van der Velde D, Ten Duis HJ, et al. Improved 1-year mortality in elderly patients with a hip fracture following integrated orthogeriatric treatment. Osteoporos Int. 2017;28:269–77	27 (6.8)	Additional treatment	Prospective cohort study	II
9	Bohl DD, Shen MR, Hannon CP, Fillingham YA, Darrith B, Della Valle CJ. Serum Albumin Predicts Survival and Postoperative Course Following Surgery for Geriatric Hip Fracture. J Bone Joint Surg Am. 2017;99:2110–8	26 (6.5)	Risk factor assessment	Retrospective cohort study	IV
10	Farrow LS, Smith TO, Ashcroft GP, Myint PK. A systematic review of tranexamic acid in hip fracture surgery. Br J Clin Pharmacol. 2016;82:1458–70	26 (5.2)	Hemodynamic management	Systematic review and meta-analysis	II

### Statistical analysis

Statistical analysis was performed with IBM SPSS Statistics software (BM Corp. Released 2018. IBM SPSS Statistics for Windows, Version 25.0. Armonk, NY, USA: IBM Corp.). Descriptive statistics (mean, SD, minimum and maximum) were computed for all metric variables.

## Results

**Fig. 2:**
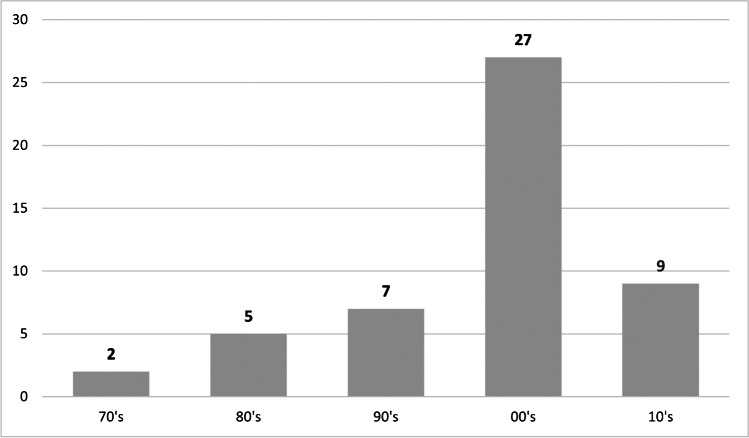
Number of studies per decade. All articles were published between 1973 and 2014 and a distinct increase was seen in the current millennium

The initial search yielded 41.782 results. In- and exclusion criteria were applied and a total of 60 articles were included in the study (Fig. 1). Based on the total citation number, a top 50 list for all time (Table 1) and a top 10 list for the years 2015 to 2020 (Table 2) were created. The top 50 articles were all published between 1973 and 2014 and a sharp increase was seen in the current millennium (Fig. 2). Thirty-eight out of the top 50 studies were published after 2000. In 2008, the most articles were published. All included studies were cited between 88 and 496 times. The mean citation density increased noticeably after the year 2000 (Fig. 2). All articles were published in English language.

### Topics

**Fig. 3:**
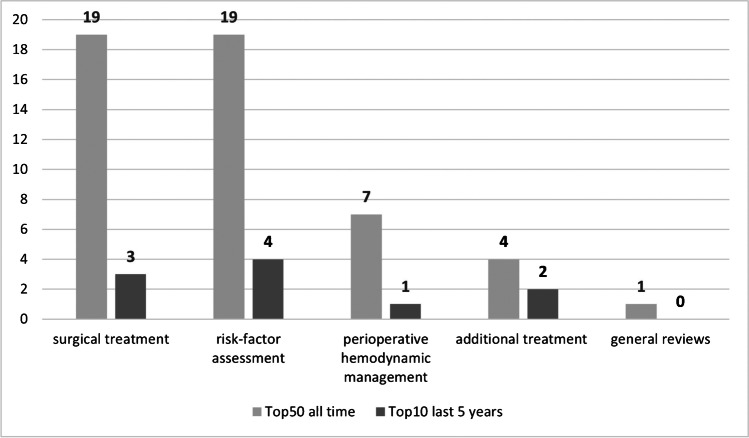
Number of studies in each topic (light grey = all time, dark grey = 2015 to 2020). Nineteen articles deal with the topic of surgical treatment, 19 with risk factor assessment, seven with peri-operative haemodynamic management, four with additional treatment and one article was a general review paper

Articles of the top 50 list covered the following topics: (1) surgical treatment (*n* = 19), (2) risk factor assessment (*n* = 19), (3) peri-operative haemodynamic management (*n* = 7), (4) additional treatment (*n* = 4) and (5) general review (*n* = 1). The top 10 studies of the last ten years addressed the same topics (Fig. 3). The assessment of risk factors for post-operative mortality included evaluation of early surgery and mobilization as well as individual parameters such as sex, age and comorbidity. Reviews regarding surgical treatment focused on different implant options such as hemiarthroplasty, total hip replacement, dynamic hip screw or cephalomedullary nails. One biomechanical study compared the helical blade with the dynamic hip screw in body donor specimens. The topic of peri-operative haemodynamic management included concerns such as hidden blood loss during surgery, thresholds for transfusion and post-operative thrombosis prophylaxis. Additional treatment options included the investigation of possible beneficial prophylaxis for osteoporosis or pre- and post-operative optimized nutrition supply. Forty-two studies investigated femoral neck fractures, 32 examined pertrochanteric fractures, two evaluated subtrochanteric fractures and in one study, the included hip fractures were not classified.

### Study type, study design and level of evidence

**Fig. 4:**
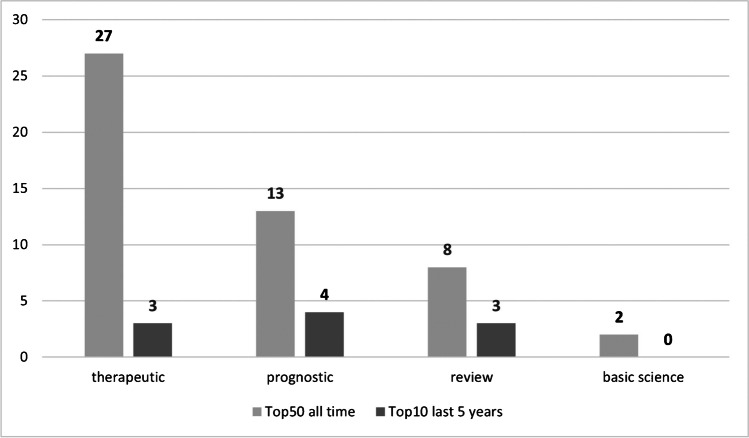
Study design of the most cited papers (light grey = all time, dark grey = 2015 to 2020). Twenty-seven articles were therapeutic, 13 prognostic, eight reviews and one basic science article

**Fig. 5:**
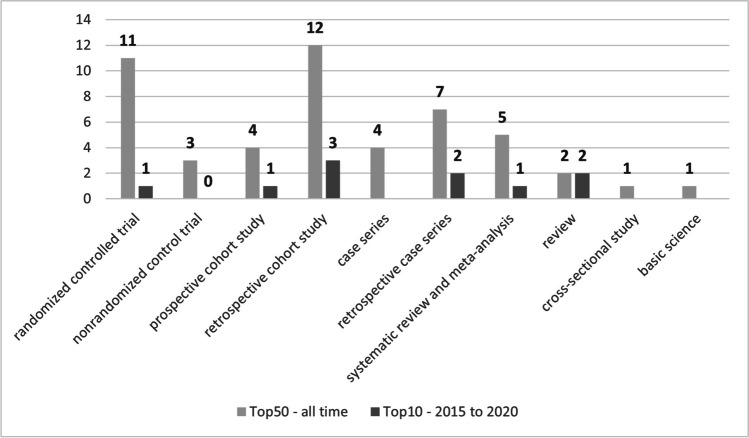
Study types of the most cited papers (light grey = all time, dark grey = 2015 to 2020). Eleven articles were conducted as randomized controlled trials, three as non-randomized controlled trials, four as prospective and twelve as retrospective cohort studies. Four were prospective and seven retrospective case series, two reviews, five systematic reviews and meta-analysis, respectively

**Fig. 6:**
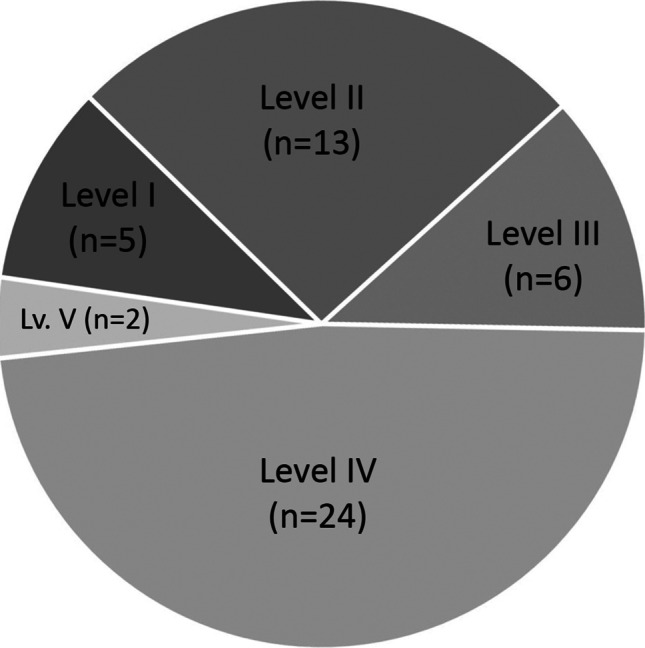
Evidence levels of the top 50 articles. Level IV was the most frequent evidence level (*n* = 24), followed by levels II (*n* = 13), III (*n* = 6), I (*n* = 5) and V (*n* = 2)

Regarding the study types, there were 27 therapeutic, 13 prognostic, eight reviews and one basic science article (Fig. 4). One study combined both basic science and therapeutic concepts. Concerning the study design, eleven articles were conducted as randomized controlled trials, three as non-randomized controlled trials, four as prospective and twelve as retrospective cohort studies. Four were prospective and seven retrospective case series, two reviews, five systematic reviews and meta-analysis, respectively. There was one cross-sectional and one basic science study (Fig. 5). Level IV was the most frequent evidence level, followed by levels II, III, I and V (Fig. 6).

### Journals and countries

**Fig. 7:**
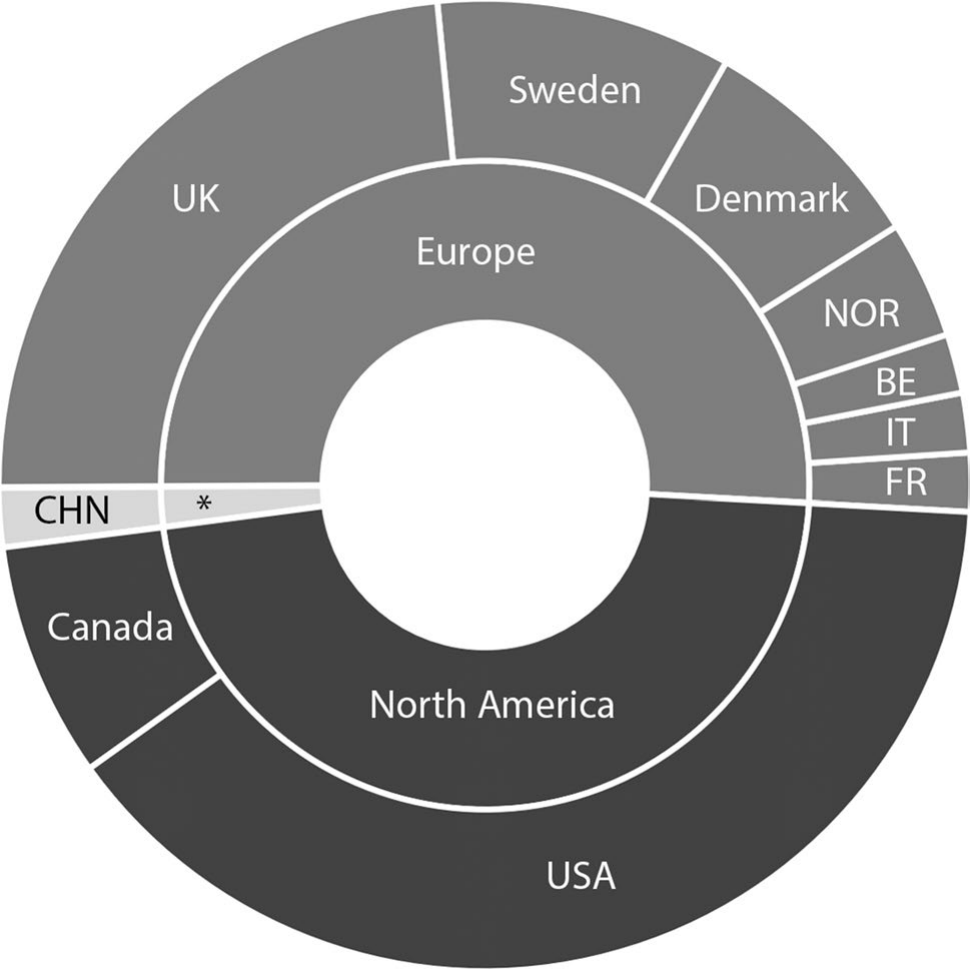
Continents and countries of origin of the Top50 most cited papers. * = Asia; CHN, China; BE, Belgium; IT, Italy; FR, France; NOR, Norway; UK, United Kingdom

The top 50 articles were published in 23 different journals. Twenty-five articles were published from institutions in Europe, 24 from institutions in North America and one from an institution in Asia. Considering the different population distribution, North America had 4.2 articles per 100,000,000 inhabitants, Europe 3.4 articles per 100,000,000 inhabitants and Asia 0.02 articles per 100,000,000 inhabitants (Fig. 7). The top ten articles from 2015 to 2020 were also primarily published in Europe (*n* = 5), North America (*n* = 4) and Asia (*n* = 1).

## Discussion

In the present study, we evaluated the most influential articles on hip fractures. The most cited papers were analyzed and a list of “citation classics” was compiled. The number one article was cited a total of 496 times and dealt with the prophylaxis of post-operative embolism in hip fracture surgery. The number of citations is comparable to the data evaluating the topic “spine fractures” [16] and “arthroscopy” [15] but stands in huge contrast to the citation numbers found on fragility fractures [18] or hip and knee arthroplasty, in which the top paper was cited 2495 times [13].

The majority of the top 50 papers focused on the optimal choice of surgical treatment (*n* = 19), followed by risk factor assessment (*n* = 19) and the peri-operative haemodynamic management (*n* = 7). The number of study topics and the enormous variation among them are not surprising, as hip fractures are the most common fractures in elderly patients, with high morbidity and mortality rates [20, 21]. Handling of these patients requires a multidisciplinary approach which includes various specialties such as orthopaedic trauma surgeons, anesthesiologists, geriatric physicians and physiotherapists. A similar collective study on spine fractures identified only two major topics, osteoporosis and pedicle screws.

**Fig. 8:**
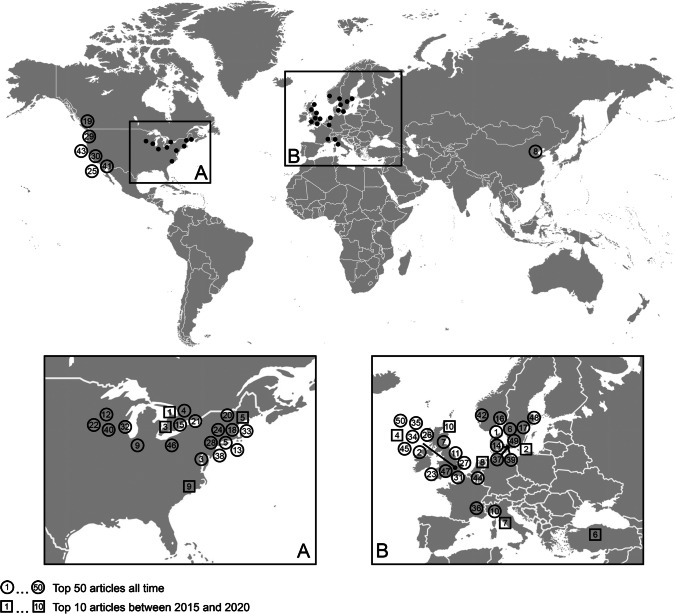
World map of the most cited papers

Most studies were conducted in Europe and North America and were written in English language. This emphasizes the huge role of these continents in scientific research and can further be supported by previous studies, in which the same countries were predominant [13, 14, 17]. A world map with the geographic areas that published research on hip fractures can be found in Fig. 8. The majority of presented articles were retrospective cohort studies (*n* = 12), followed by randomized controlled trials (*n* = 11) and retrospective case series (*n* = 7). Randomized control trials, the gold standard of scientific research, are almost evenly distributed among studies of the optimal choice of surgical treatment and peri-operative haemodynamic management. The large number might be due to the high incidence of hip fractures, which favours big trials. This contrasts similar studies on hip arthroscopy or fragility fractures, where randomized controlled trials were the minority and case series were prevailing [14, 18]. Lefaivre et al. could show that among the top 100 articles published in the field of orthopaedics, there was not a single randomized controlled trial [22]. This can be explained by the study design and the wide range of orthopaedic subspecialties and their individual level of knowledge. In the article about spine fractures, no absolute numbers are given about the type of study [16].

There has been an increase in studies and mean citation densities since 2000 (Fig. 2). This finding is consistent with previous studies on spine fractures and represents the knowledge gain of the last 20 years [16]. Data in the present study clearly demonstrates the increase of studies on haemodynamic management and additional treatment in the last 20 years, as physicians became aware of the importance of individual and adjuvant treatment (i.e. osteoporosis treatment) after surgery, similar to the study from Donnally et al. concerning spine fractures [16]. Hip fractures are life-threating events due to the various pre-existing conditions of this geriatric and multimorbid patient population. The surgery itself is not solely responsible for the patient’s survival and outcome [23]. This may be further supported by the increasing age of patients and the increasing understanding of the molecular biology parameters of osteoporosis. This implication is further accompanied by a higher evidence level over the last 20 years. All level I studies of the top 50 articles were performed between the years 2003 and 2010. However, level IV was still predominant overall. Present distribution is similar to recent articles on spine fractures, hip arthroplasty or hip arthroscopy [13, 14, 16].

There are some intrinsic problems with this kind of study and citation analysis. As previously described, it does not account for self-citation and the author’s preference to cite articles in the journal in which they seek to publish their own work [13–15, 17, 24]. Furthermore, there is a clear time effect in citation analysis. The most recent articles are at disadvantage, because there is not enough time for citations to accumulate. To compensate for that, we included the top ten articles of the last five years. Another possible weakness is the “Snow-Ball” effect, which suggests that authors are likely to cite a study that a previous publication has cited without questioning the quality and accuracy of this study [25]. The total citation count was used to determine the ranking, because we wanted to show the exponential growth medical research has experienced during the last decades. However, we added the citation density in Tables 1 and 2 for a better understanding of the article´s impact. Finally, we recognize that in many contexts, the value of a contribution cannot be quantified simply by the number of citations a publication receives.

We created a top 50 list of citation classics and elucidated the change of research focus during the last 20 years. The top ten list from 2015 to 2020 was added to highlight the most recent topics. In conclusion, the most cited studies on hip fractures focused primarily on surgical treatment options and risk factor assessment. After the year 2005, studies about additional treatment options for osteoporosis and an optimal post-operative care gained in importance. The presented tables with references can serve as a guide for a comprehensive understanding of the historical and current literature pertaining to hip fractures.

## Data Availability

The authors confirm that the data supporting the findings of this study are available within the article. Additional data is available from the corresponding author (Madeleine Willegger) on request.
